# Anomalous right coronary artery with interarterial course depicting an unusual case of an electrical storm: a case presentation

**DOI:** 10.1186/s12872-020-01486-1

**Published:** 2020-04-22

**Authors:** Snehasis Pradhan, Kciku Gresa, Hans-Joachim Trappe

**Affiliations:** Department of Cardiology and Angiology, Marien Hospital Herne, Ruhr- University of Bochum, Hoelkeskampring 40, 44625 Herne, Germany

**Keywords:** Anomalous RCA, AAORCA, Case report, Antiarrhythmic drugs, Electrical storm, CRT, Surgical intervention

## Abstract

**Background:**

The condition of anomalous aortic origin of the right coronary artery (AAORCA) with an interarterial course leads to few, if any, clinical problems. Malignant presentation of the often non-significant AAORCA associated with conduction system abnormalities is a rare finding. Surgical repair, even for highly symptomatic patients, is still controversial. However, in this case, the surgery brought a paradigm shift in treatment modality, improving the symptoms of this patient.

**Case presentation:**

We report a case of a 52-year-old man with severe chest pain and recurrent electrical storms with an implanted cardiac resynchronization therapy defibrillator (CRT-D) device. Coronary angiography and computed tomography (CT) revealed the rare anomalous aortic origin of the right coronary artery (AAORCA) with a high interarterial course between the aorta and the pulmonary trunk. As symptoms typically develop on exertion, placing the patient at an increased risk of ischemic distress, a stress myocardial perfusion imaging study helped to identify his high-risk status. Although patient-specific, a surgical repair was the only concrete step agreed upon after multiple collaborative discussions with the cardiac surgeons. Surgery significantly improved the symptoms, with the patient reporting resolution of his chest pain, as well as no documented inappropriate defibrillator activity on follow-up appointments.

**Conclusion:**

One purpose of reporting the case study was to underscore the risk factors associated with AAORCA, challenging claims of its benign nature. This case complements existing findings demonstrating that ischemic distress consequent to the right coronary artery (RCA) compression may precede the rare incidence of an electrical storm. Importantly, the case-study emphasizes the significance of integrated multimodality imaging in clinical practice as well as providing real-world evidence for the efficacy of surgical repair in highly symptomatic patients with AAORCA with an interarterial course.

## Background

Anomalous aortic origin of the right coronary artery (AAORCA) from the contralateral sinus of Valsalva with a high interarterial course between the aorta and the pulmonary trunk is a rare phenomenon, often an incidental finding, which has a prevalence rate varying between 0.026 and 0.25% [1]. AAORCA has been reported more frequently than the anomalous aortic origin of the left coronary artery (AAOLCA) [2] and is often thought as clinically insignificant; some, however especially in young patients, have been associated with an increased risk of sudden cardiac arrest or death, myocardial ischemia, arrhythmia and syncope. Most coronary anomalies are second only to hypertrophic cardiomyopathy as a leading cause of sudden cardiac death in young athletes. The pathophysiology of sudden death in this entity is unclear and has not been thoroughly elucidated to date.

Based on the literature review, anomalous RCA may be broadly classified into three variants; A high interarterial course between the aorta and the pulmonary artery, a low interarterial course between the aorta and right ventricular outflow tract and a hypoplastic anomalous RCA orifice with a significantly reduced arterial course.

The prevalence of typical angina and that of major adverse cardiac events (MACEs) as described by Lee et al. was significantly higher in patients with high interarterial course as compared to low interarterial course [2]. Due to their rare incidence, physicians may misdiagnose these events as typical ischemic conditions. Risk stratification is frequently determined through myocardial functional studies to assess for evidence of inducible ischemia. Ischemic disturbances may lead to clinical presentation of electrical storm (also known as sympathetic storm) that can often be dramatic in nature and may be a consequence of implanted cardioverter-defibrillator, heart diseases, arrhythmic syndrome, myocardial infarction or – as seen in the present case – myocardial ischemia. Effective management of electrical storm requires knowledge of the mechanisms of arrhythmias. In most patients, amiodarone and beta-blockers are the best therapeutic options for the treatment of arrhythmia [3].

Surgical intervention is controversial in the case of RCA anomalies because there are few clinical symptoms. Of particular note, in highly symptomatic patients presenting with recurrent ischemia or ventricular arrhythmia, a surgical repair can be accomplished with relatively few postoperative complications and can lead to drastic resolution of the symptoms [4]. In the current case report, retrospective analysis has been carried out on the emergency rescue of AAORCA associated electrical storm and relevant literature were reviewed.

## Case report

A 52-year-old man presented in the emergency department of our hospital with a history of typical angina and recurrent electrical storms with an in situ cardiac resynchronization therapy defibrillator (CRT-D) device, implanted 9 years ago.

The patient’s medical records revealed that an emergency coronary angiography had been performed 11 years ago for a transient posterior wall myocardial infarction. Interestingly no culprit lesion was discovered, with simultaneous left ventriculography showing no significant LV-function impairment. Two years later, the patient was readmitted with severe dyspnea and recurrent angina. A repeat coronary angiography could not establish an ischemic cause of the symptoms in the absence of any relevant coronary artery stenosis. Echocardiography showed a dilated left ventricle with severely reduced left ventricular ejection fraction (LVEF = 28%). Further investigation with cardiac-MRI, in the absence of any extensive myocardial scarring and specific tissue characteristics, excluded probable ischemic, dilated, hypertrophic, restrictive and arrhythmogenic aetiologies of severely reduced left-ventricular function. The precise pathophysiology governing the heart failure and ventricular remodelling remained in this case obscure. Hence, a provisional diagnosis of idiopathic nonischemic cardiomyopathy was proposed.

In view of continuing symptoms despite optimal heart failure regimen and without further improvement of left ventricular function, a primary prevention strategy with programmed ventricular stimulation was adopted. The patient underwent conventional CRT-D system implantation in the presence of a complete left bundle branch block (LBBB).

Remarkably, the patient was a classic CRT-D responder with the improvement of LVEF up to 42% with no unusual electrical activity over the period. As part of a continuous heart care program, the patient had been attending regularly scheduled cardiology follow-up consultation. The patient showed to date no further deterioration of his cardiac function and had no clinical episodes of VT or shocks; however, he continued to have intermittent angina throughout. None of the further records revealed any ischemic cause of symptoms, except one of the angiograms showing the non-traceability of RCA.

**Fig. 1:**
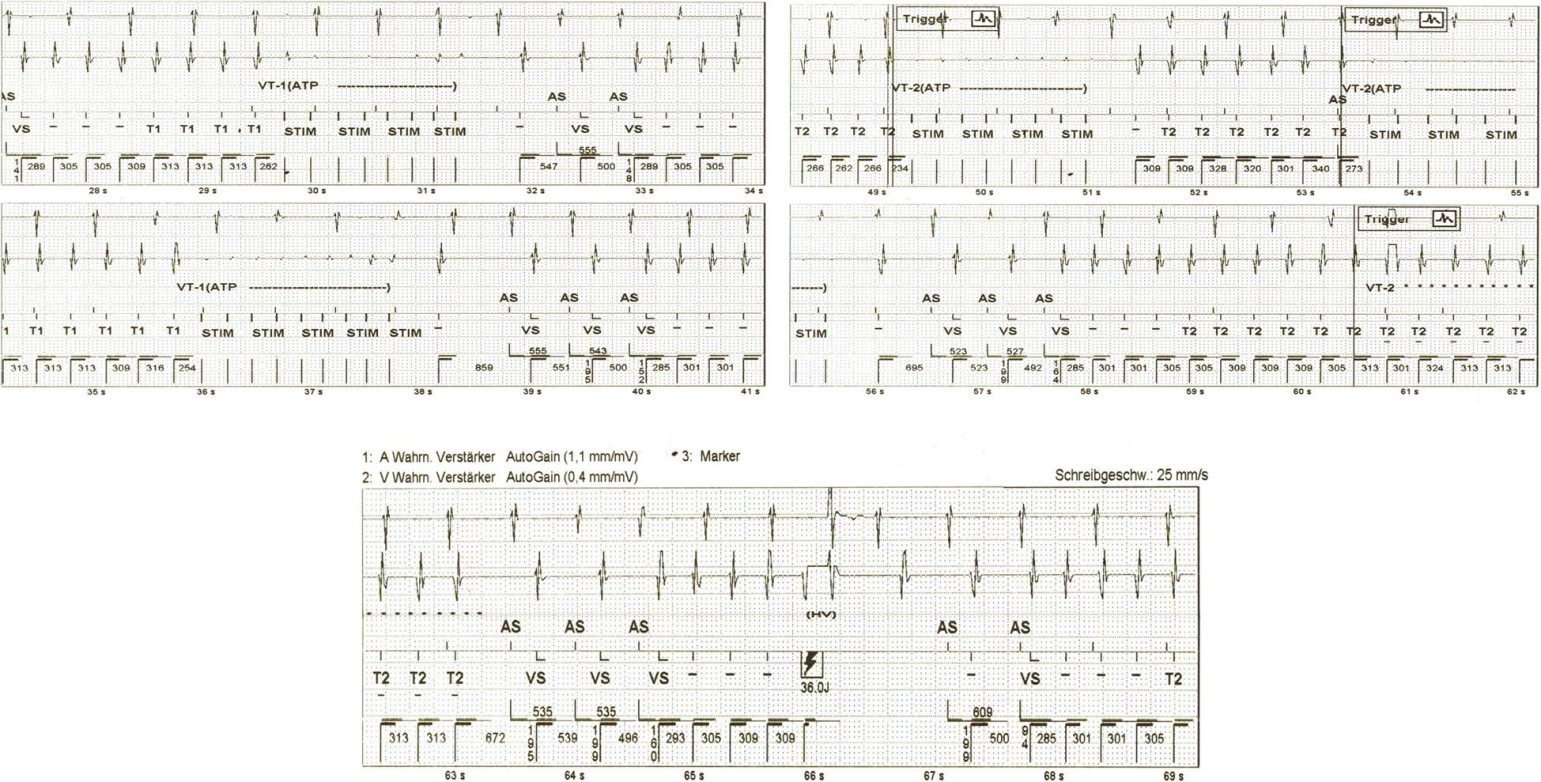
Endocardiac electrograms recorded by CRT-D demonstrating the unsuccessful ATP therapy to Ventricular Tachycardia (VT1 and VT2) episodes and successful high-energy shock therapy

Nine years after the implantation of CRT-D, the patient presented to us experiencing electrical storm for the first time, with a total of 22 malignant ventricular tachyarrhythmic episodes that were recorded in the tachycardia zone (Fig. 1). After a few unsuccessful attempts at anti-tachycardiac pacing (ATP), the device delivered a total number of 25 appropriate maximal energy shocks at 38 J following ongoing tachycardia episodes.

**Fig. 2:**
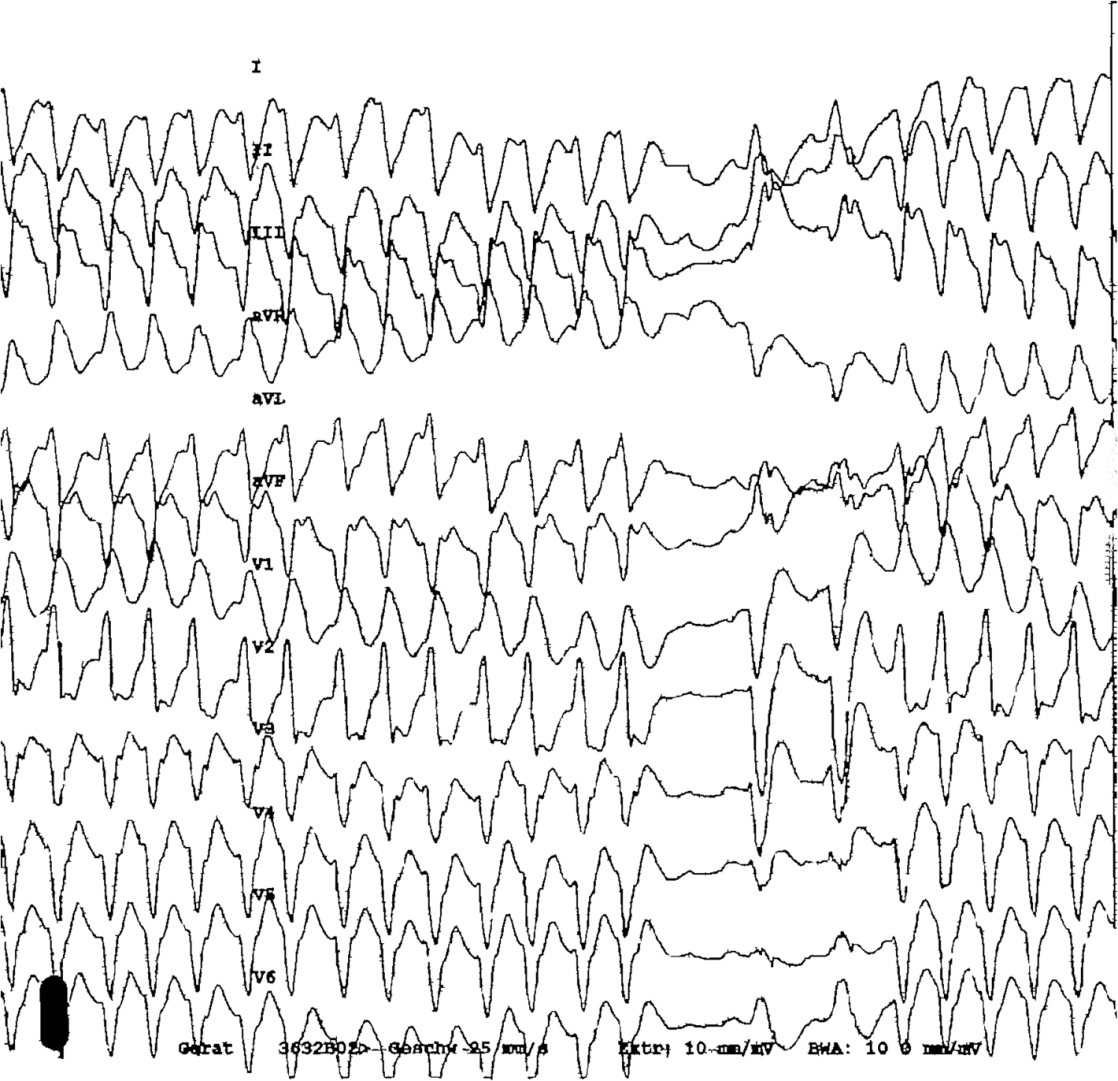
12-lead electrocardiogram showing ventricular tachycardia with a right bundle branch RBBB-like morphology, an early precordial transition at V3 and predominant S in aVF

**Fig. 3:**
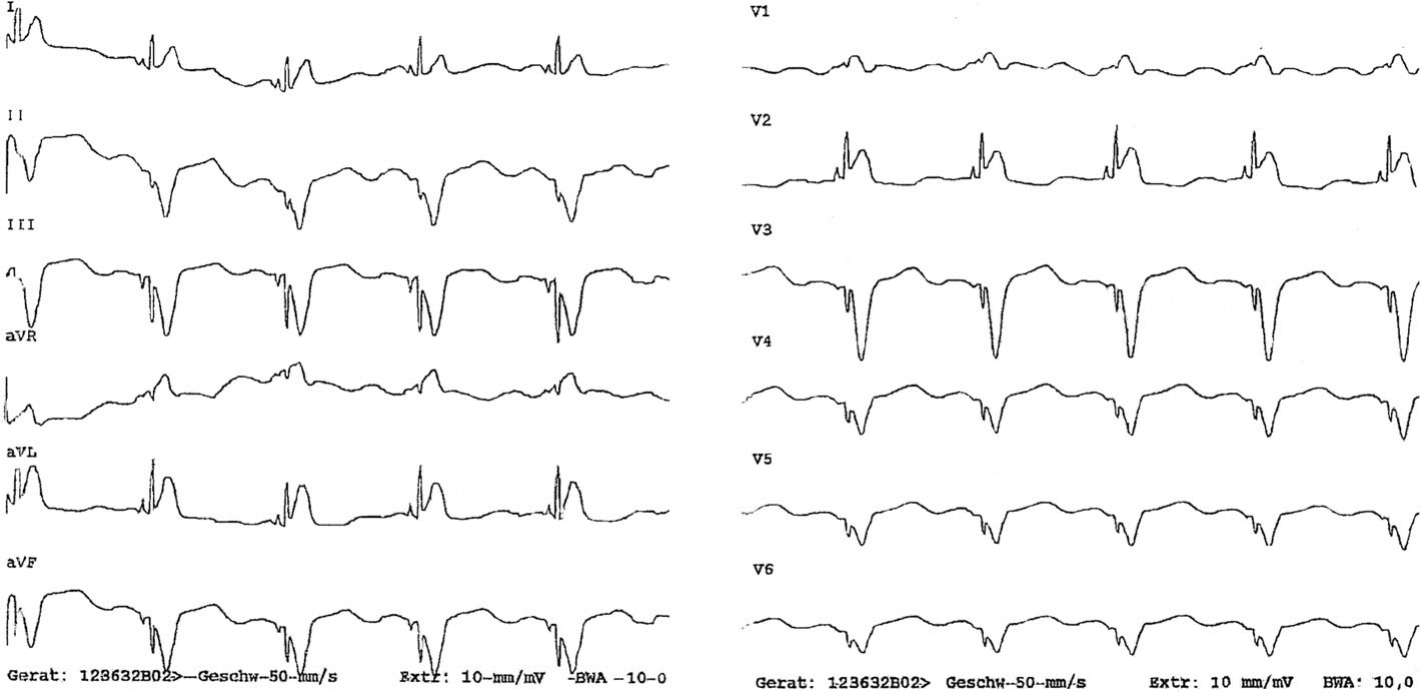
12-lead electrocardiogram showing a ventricular paced rhythm without any relevant concordant ST elevation (“negative” Sgarbossa criteria)

A 12-lead electrocardiogram (ECG) (Fig. 2) at presentation showed ventricular tachycardia (VT). The patient was treated with 300 mg intravenous amiodarone bolus, which subsequently terminated the electrical storm. Later, 1000 mg/day in three divided doses, oral amiodarone was administered to establish the desired plasma level of the drug. An acute ST-Elevation Myocardial Infarction (STEMI) was subsequently excluded in a follow-up 12-lead ECG showing ventricular-paced rhythm without any relevant concordant ST elevation (“negative” Sgarbossa criteria) (Fig. 3).

Further physical examination revealed that our patient was highly anxious and dyspneic with an initial fluctuating heart rate of over 200/min and blood pressure of 126/80 mmHg. The patient denied any recent episode of syncope. On pulmonary auscultation, bilateral basal crepitations were audible, which was later confirmed by chest X-ray as bilateral pulmonary congestion. The cardiovascular risk factors associated with the patient were hypertension, history of smoking (around 20 cigarettes/day) and elevated body mass index (BMI = 37).

Initial laboratory test results showed normal levels of serum potassium, magnesium and thyroid stimulating hormone; however, the levels of creatine phosphokinase and creatine phosphokinase (MB) were elevated, i.e. 671 IU/L and 41 IU/L, respectively (optimal range CK 0–174 and CK-MB 0–25 IU/L). High-sensitivity troponin assays revealed a staggering increase from 41 pg/ml to 264 pg/ml within 2 h of the initial presentation (optimal range 0–14 pg/ml). The echocardiography and other laboratory investigations did not support left ventricular hypertrophy, acute deterioration of cardiac function (ejection fraction = 40%), electrolyte disturbances or any other triggering factors as an etiology for arrhythmic storm and acute coronary syndrome (ACS).

**Fig. 4:**
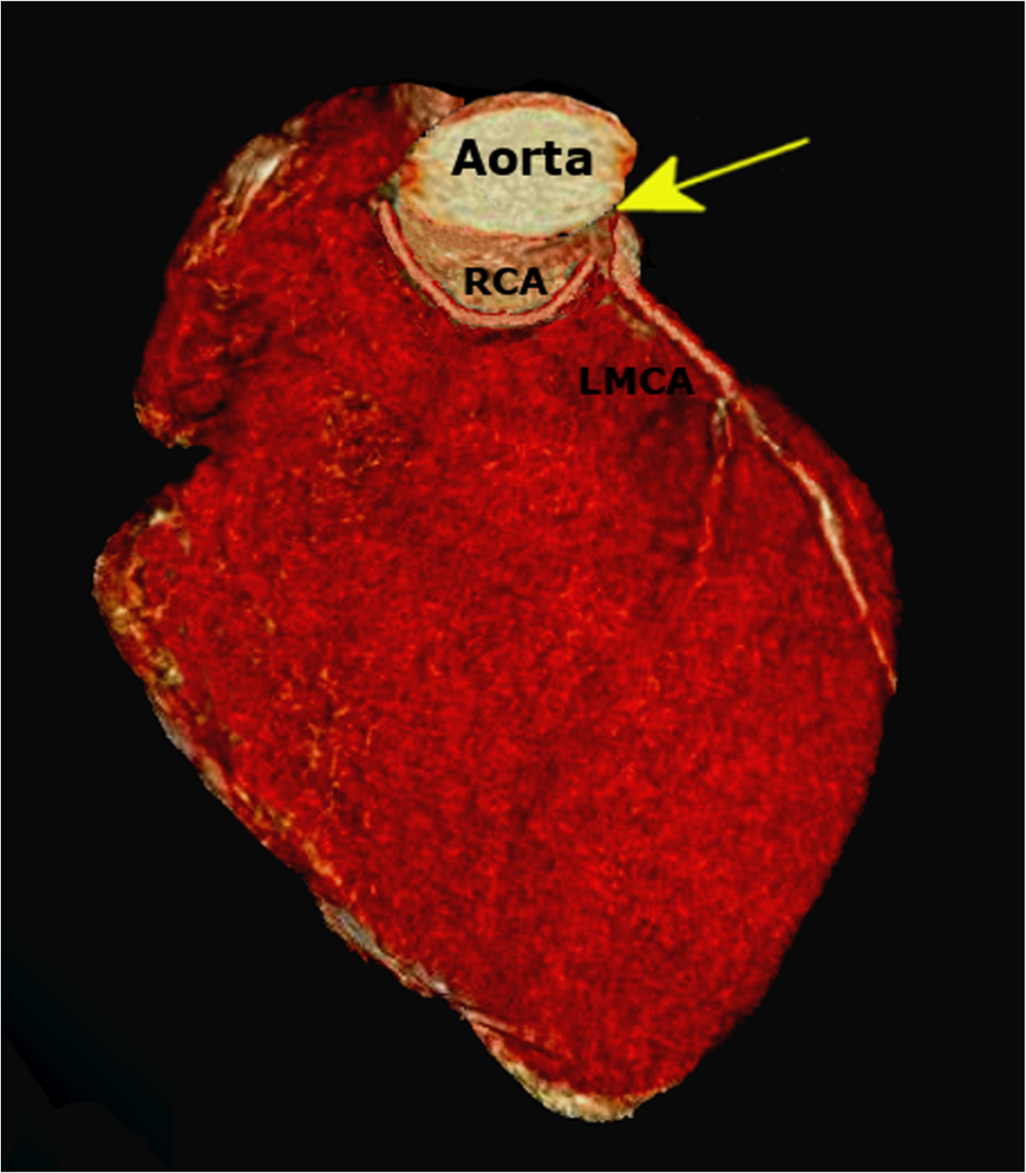
Computed tomography (CT)-based three-dimensional (3D) reconstruction showing the origin of RCA from the left sinus of Valsalva (arrow). The normal origin of the left main coronary artery (LMCA) is also seen

**Fig. 5:**
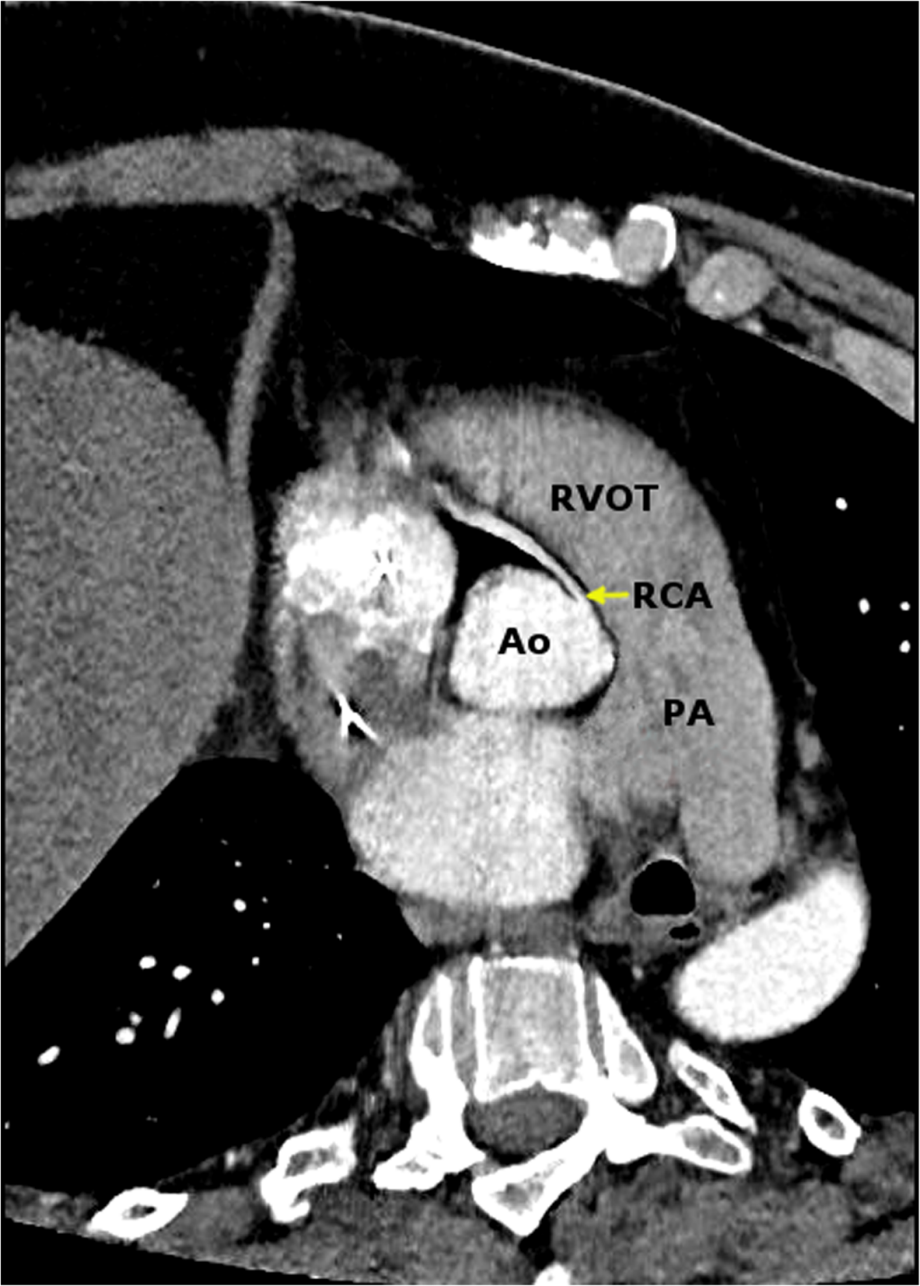
Computed tomographic angiogram indicating compression of RCA between the great vessels at its origin. (PA-Pulmonary artery, RVOT- Right ventricular outflow tract, Ao-Aorta)

Based on his history of continued symptoms and increased level of cardiac enzymes, the patient underwent immediate catheterizations. The diagnostic coronary angiogram excluded the presence of any culprit lesion, but revealed a non- significant 30–40% stenotic multivessel coronary disease of left anterior descending artery (LAD) and left circumflex (LCX) with normal TIMI III flow pattern. Anatomically, the left main coronary artery was observed to originate from the left aortic sinus. With the conventional angiogram using a right Judkins catheter after several attempts at cannulation, the course of the RCA was not traceable. Hence it was followed by the computed tomographic (CT) coronary angiogram, which revealed an RCA originating from the left coronary sinus (Fig. 4) with an interarterial course in between the aorta and pulmonary artery leading to dynamic compression with evident decreased luminal diameter at the origin of RCA between the great vessels without any distinct narrowing of the remainder of the RCA (Fig. 5).

Based on the initial ECG morphology of VT (Fig. 2) with a right bundle branch block RBBB-like morphology, an early precordial transition at V3 and predominant S in aVF; the wavefront of VT was therefore thought to exit and activate in the inferior aspect of the left ventricle. Subsequently, we urged for an electrophysiology study, to which the patient did not give consent. Cardiovascular surgical evaluation of the interarterial compression of RCA excluded the need for any immediate surgical intervention due to the lack of concrete evidence of any myocardial ischemia.

Over the next 12 h, the patient’s clinical status improved without any recurrence of arrhythmia. The heart failure regimen was optimized with increased dosage of the β-blocking agent (metoprolol) and ACE inhibitor (ramipril) along with the addition of an aldosterone antagonist (eplerenone). We proposed the new ARNI (Angiotensin receptor- Neprilysin inhibitor) as an alternative to an ACE inhibitor as per the current heart-failure guideline, but the patient denied this therapeutic proposition. After close monitoring for any adverse effects, the patient was finally discharged on a maintenance dose of 200 mg/day of amiodarone.

**Fig. 6:**
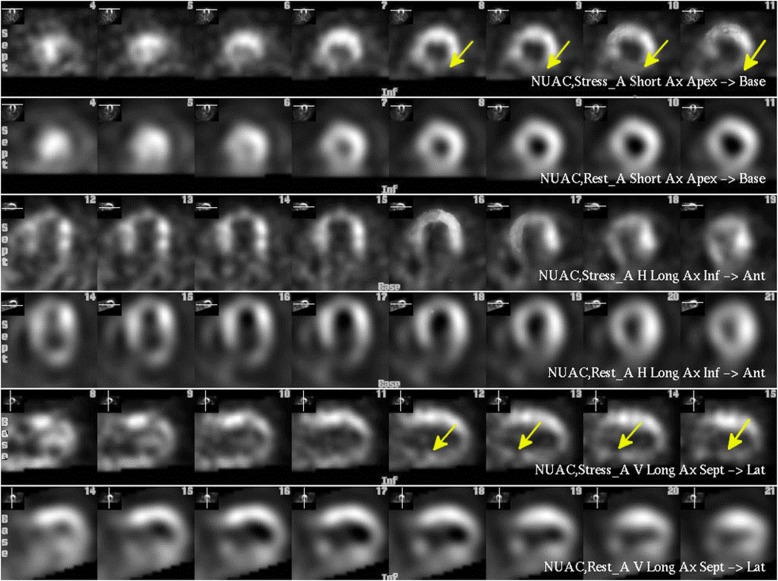
Myocardial perfusion SPECT scintigraphy demonstrating the short axis (SA) slices (top), vertical long axis (VLA) slices (middle), and horizontal long axis (HLA) slices (bottom). The top row of each pair is at stress and the bottom at rest. The yellow arrows show the perfusion defect in the inferior wall under physiological stress

The patient continued to experience persistent angina at rest, for which he was again hospitalized after 3 weeks. His condition prompted an ischemic evaluation to determine the extent of the myocardial perfusion. Radionuclide imaging using myocardial perfusion scintigraphy (MPS) was performed, which revealed extensive reversible perfusion defect involving the inferior and posterior wall coinciding with myocardial territory supplied by the RCA (Fig. 6).

In the absence of any culprit lesion along with MPS based preliminary evidence of delineated ischemic area consistent with the VT exit site on 12-lead ECG, we hypothesised that the ventricular arrhythmia and severe angina episodes could be due to the atypical anatomy of the RCA with an interarterial course. Hence, we referred the case for surgical re-evaluation. After a thorough discussion, the patient was transferred to the cardiothoracic department for surgical unroofing, believing that the ischemia was due to interarterial dynamic compression of RCA. The patient was followed-up for 10 months after surgery; he remained asymptomatic without any further episodes of angina pectoris or electrical storms.

## Discussion and conclusion

The present case study highlights a strategy for the management of ischemic distress without any notable coronary culprit lesion. The case is of particular interest because it spans from the assessment of coronary vessels following an electrical storm until the evaluation of a surgical repair several weeks later.

At the presentation under discussion, the patient experienced an electrical storm with 25 appropriate shocks. Electrical storm usually results in hypotension and symptoms of weakness, syncope, or palpitations [5]; however, in the present case report, the onset of arrhythmia was evident with the patient being normotensive without any syncopal episode. Further interrogation of the device showed neither any inappropriate programming error (including T-wave oversensing or R-wave undersensing) nor any electrode dislocation. Thus, it was concluded that the CRT-D was effective in interrupting malignant arrhythmias.

Although coronary angiography could not establish any immediate ischemic cause for the electrical storm, radionuclide imaging showed ischemia in the posteroinferior wall indicating a decreased flow in the RCA matching to apparent VT exit site on 12 lead ECG. Stress tests, however, are not always positive for inducible ischemia in this cohort of patients [6, 7]. Although an electrophysiology study might have provided further insight into the origin of ventricular tachycardia in this case, we still would not have established a direct relationship between RCA compression and electrical storm.

It is conceivable that triggering of the arrhythmic episode might have been influenced by other factors such as impaired left ventricular function and the potentially pro- arrhythmic nature of CRT-D itself [8]. However, the fact that the patient was a super CRT-D responder along with the event-free period of nine consecutive years after the device implantation was evidence against these factors having a primary role in triggering the electrical storm [9]; although this cannot be ruled out completely.

We assumed that sustained ischemia leading to recurrent angina – and, as in this case, the electrical storm – could have occurred due to the compression of the right coronary origin between the aorta and pulmonary artery. Alternatively, asymptomatic ventricular arrhythmias because of AAORCA might be one of the dominant pathologies behind the cardiomyopathy. However, the implantation of CRT-D, along with optimal heart failure therapy, did offer some protection against serious ventricular arrhythmia burden for a prolonged period until the manifestation of electric storm.

In our case, the patient’s young age was a significant factor in his poor adjustment to severe cardiac illness. The incidence of shock therapies delivered by the device was high and interfered with the quality of his life. This cohort of patients often have expectations for their quality of life, that may be appropriate for their age, but not for their level of health, resulting in heightened frustration and psychological distress [10], which seems to be the reason for the non-compliance of our patient at multiple stages.

In conclusion, the association of electric storm with the interarterial course of RCA is an extremely rare but potentially malignant presentation. The choice of treatment for such a cardiac anomaly is controversial, but these clinical attributes may make a case for surgical repair as the most rational treatment modality.

**Fig. 7:**
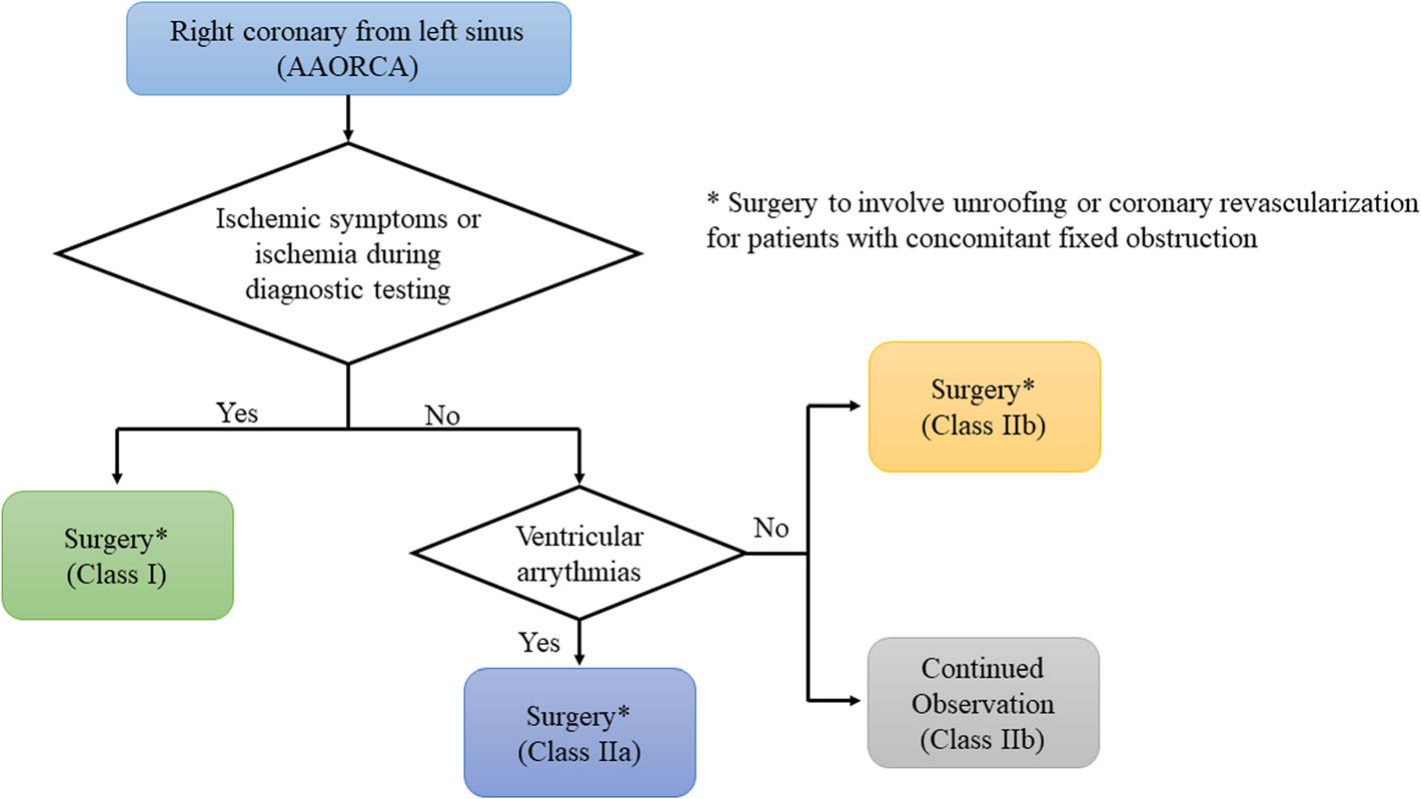
Algorithm for management of anomalous aortic origin of right coronary artery taken from 2018 AHA/ACC Guideline for the Management of Adults With Congenital Heart Disease

The surgical unroofing of AAORCA is a novel approach in these patients and was performed in our case due to persistent angina, high course of the AAORCA and evidence of external compression of the coronary artery. This surgical intervention is supported by the fact that even a small amount of physical exertion may lead to an expansion of the aortic root and pulmonary trunk, increasing the existing angulation of the right coronary artery and narrowing the luminal diameter [11]. Hence, the unroofing procedure manipulates the orifice, enlarges the orifice, and makes an acute angulation, which decreases the lateral compression of the intramural segment [12]. 2018 AHA/ACC guideline for the management of adults with congenital heart disease [13] recommends surgery in AAORCA for symptoms or diagnostic evidence consistent with coronary ischemia attributable to the anomalous coronary artery (class I recommendation), whereas surgery for AAORCA in the setting of ventricular arrhythmias is seen to be as reasonably beneficial in the treatment algorithm (Fig. 7).

A study done by Angelini et al. [14] suggests that the dynamic compression of anomalous RCAs should be quantified by intravascular ultrasonograms using a custom-made guiding catheter, explicitly designed to coaxially access the ectopic and tangential RCA. Percutaneous coronary intervention (PCI) may then be used in critical stenosis to relieve systolic compression.

The benefit of excessive exercise limitation is doubtful, and in general, it is only recommended for patients who are awaiting surgical intervention, who are currently in the postoperative period, who have high-risk lesions and refuse surgical intervention, or who have ischemic symptoms or positive functional testing but whose anatomy is unsuitable for surgical intervention. Patients with low-risk lesions that do not warrant surgical intervention are not exercise restricted [7].

The present case underscores the following clinically essential points:

1. Frequently, at catheter angiography, because of its complex three-dimensional geometry, the precise course of the anomalous vessel may be difficult to delineate in two dimensions fluoroscopically. Difficulties with selective cannulation of the anomalous vessel may lead to the erroneous assumption that the vessel is occluded. Hence, further confirmatory exploration is warranted for suspicion of RCA anomaly. Integration of multimodality imaging like coronary CT angiogram can provide an excellent insight into the precise anatomy and course of the anomalous coronary artery, including ostial morphology.
2. An AAORCA from left coronary sinus can have dynamic narrowing and kinking, causing symptoms of myocardial ischemia, malignant heart rhythms and appropriate shock episodes with defibrillator devices, and may also lead to sudden cardiac death.
3. A proper classification of patients in this cohort, based upon the symptoms and level of risks (low to high), can guide close monitoring and follow up these patients. It is essential to include a radionuclide imaging in the follow-up diagnostics for better evaluation of myocardial perfusion. However, nuclear scans can lead to false positive, especially in the young population.
4. Assisting poorly therapy-compliant patients with psychological interventions can help them to allay any unrealistic fears and to potentially achieve a better disease prognosis.

## Data Availability

Not applicable.

## Abbreviations

AAORCA: Anomalous aortic origin of the right coronary artery
AAOLCA: Anomalous aortic origin of the left coronary artery
ATP: Anti-tachycardiac Pacing
ACS: Acute coronary syndrome
BMI: Body mass index
CRT-D: Cardiac resynchronization therapy defibrillator
CT: Computed tomography
LVEF: Left ventricular ejection fraction
MPS: Myocardial perfusion scintigraphy
MACEs: Major adverse cardiac events
LBBB: Left bundle branch block
RBBB: Right bundle branch block
STEMI: ST-Elevation Myocardial Infarction
VT: Ventricular tachycardia
